# The evaluation of an integrated network approach of preventive care for children with overweight and obesity; study protocol for an implementation and effectiveness study

**DOI:** 10.1186/s12889-019-7304-1

**Published:** 2019-07-23

**Authors:** Sanne de Laat, Iris de Vos, Monique Jacobs, Edgar van Mil, Ien van de Goor

**Affiliations:** 1 School of Social and Behavioural Sciences, Tranzo, Tilburg University, Postbus 90153, 5000 LE Tilburg, The Netherlands; 2 GGD Hart voor Brabant, Postbus 3024, 5003 DA Tilburg, The Netherlands; 3 GGD West Brabant, Postbus 3024, 5003 DA Tilburg, The Netherlands; 4 Jeroen Bosch Hospital, Department of Paediatrics, Postbus 90153, 5200 ME ‘s-Hertogenbosch, The Netherlands

**Keywords:** Overweight, Obesity, Children, Network, Central care provider, Integrated care, Effectiveness, Implementation, Quality of life, Youth health care (3–10 keywords)

## Abstract

**Background:**

Children with overweight often do not receive appropriate integrated care. An innovative integrated network approach of preventive care for overweight children aged 4–12 years old has been developed and implemented in four neighbourhoods of ‘s-Hertogenbosch, The Netherlands. This new approach focusses on self-management of the family and is based on the principles of stepped and matched care. Youth health care (YHC) nurses support the families in their new role as central care providers. The aim of this study is to evaluate the implementation and effectiveness of this network approach.

**Methods:**

The implementation of the new approach (reach, functioning of the central care provider, network functioning and patient satisfaction) is assessed by interviews and checklists with professionals and parents of 4–12 year old overweight or obese children. To evaluate effectiveness, we aim to compare 120 overweight or obese children in ‘s-Hertogenbosch with 60 overweight or obese children outside ‘s-Hertogenbosch during one year of YHC involvement. Quality of life, psychosocial problems of the child and parental empowerment are the main outcomes of the effectiveness study. Outcomes are measured with digital questionnaires at inclusion, at three months and one year after inclusion. BMI measurements and referrals are distracted from medical files.

**Discussion:**

Integrated care for overweight and obese children is high on the agenda of many municipalities in The Netherlands. The new approach is expected to have beneficial effects for overweight children, their parents and professionals. With the results of this study, we can optimize the support for overweight and obese children and their parents. The first results are expected to be available in 2019.

**Trial registration:**

This study is registered in the Dutch Trial Register on 10 November 2017 (NTR number NTR6813). https://www.trialregister.nl/trial/6596

Word count: 281 (max 350).

## Background

Overweight is a considerable health problem for the Dutch society as it is for most countries in North-western Europe [1]. In 2017, almost half of the adult population in the Netherlands was overweight and 13.9% was obese [2]. The percentages for children between 4 and 11 years old were lower, respectively 13.1 and 3.3% [3].

The consequences of overweight and obesity are not to be underestimated. Childhood overweight and obesity have been associated with several physical problems on the short- and long-term [4]. Children have a higher risk of glucose intolerance, joint problems, diabetes mellitus type 2, hypertension, hypercholesterolemia and sleep apnoea [5–7]. In addition, overweight is also associated with low self-esteem, bullying, depression, loneliness, and sadness [8–11]. Furthermore, studies showed that overweight children are prone to stay overweight in adult life, which is a risk factor for various chronic diseases [12, 13].

The prevention of overweight and obesity at a young age is of high priority for the Dutch government and municipalities. Overweight prevention is embedded in the National Prevention Agreement, which is part of the governmental coalition agreement of 2017–2021 [14]. Although the importance of the prevention of childhood overweight and obesity is commonly acknowledged, current preventive care for children with overweight and obesity is not optimal. The (preventive) care is fragmented, not optimally aligned and provided by various health care professionals. Collaboration between professionals and the coordination of care can be improved [15–17].

Therefore, an innovative model for an integrated network approach of preventive care for children between 4 and 12 years old has been developed and implemented in four neighbourhoods in ‘s-Hertogenbosch, The Netherlands. Within this innovative approach, overweight is not primarily seen as a consequence of an unhealthy diet and insufficient physical exercise, but rather as a symptom of underlying problems [18]. The new approach focusses on self-management of the family, is based on principles of stepped and matched care and the self-determination theory [19]**.** Health care professionals of both the medical and social domain work together in close collaboration and according to the same principles and methods as described by Van Mil & Struik [18, 19]. The care provided by the professionals is coordinated by a central care provider. In ‘s-Hertogenbosch the role of central care provider is assigned to youth health care (YHC) nurses. YHC professionals (YHC nurses and YHC doctors) see all children between 0 and 18 years old at set ages. The YHC is provided free of charge and is voluntary. More than 95% of the children are regularly seen at a YHC centre, where their height and weight is measured [20]. Moreover, the YHC nurses are positioned as linking pins in the multidisciplinary network of local professionals, both within the medical and in the social domain. So, they seem to be an appropriate professional for the role of central care provider for overweight and obese children.

The aim of this study is to evaluate the implementation and effectiveness of the integrated network approach of preventive care for children with overweight and obesity in ‘s-Hertogenbosch, in which YHC nurses fulfil the role of central care providers. It is expected that with this new approach more children and parents will be reached and actively followed up. Moreover, by matching the care with the needs of parents and children and through the optimal use of strong local networks, we expect to achieve lasting effects on quality of life for overweight and obese children.

## Methods

### Design

**Fig. 1:**
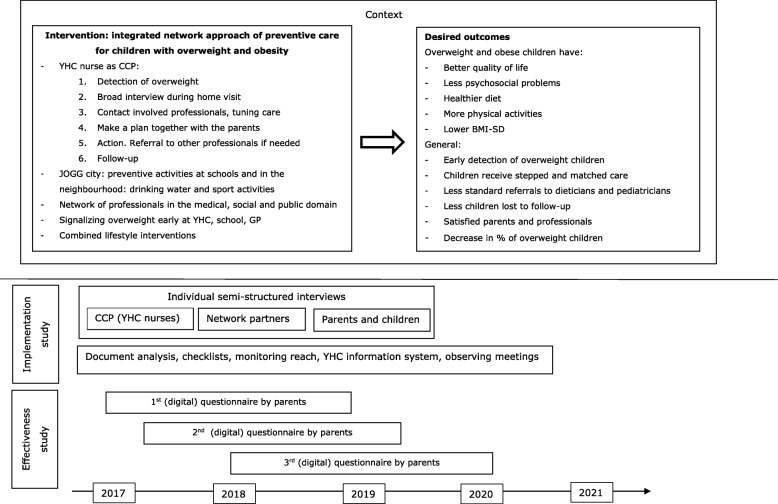
Overview of the study

In this mixed-methods study we evaluate the processes and effectiveness of the integrated network approach of preventive care for overweight and obese children in ‘s-Hertogenbosch, using a longitudinal quasi-experimental design. Quantitative and qualitative data are used to answer the research questions. The study is divided in an implementation and an effectiveness study. Data will be collected from October 2017 till January 2020. The study protocol has been approved by the Medical Ethics Committee Brabant (reference number METC P1737). An overview of the study can be found in Fig. 1.

#### Implementation study

In the integrated network approach the role of the YHC nurse as central care provider is crucial. To study the extent to which YHC nurses fulfill their tasks of central care provider and the factors associated with their ability to fulfill these tasks, semi-structured interviews are held with all YHC nurses in the neighbourhoods. Furthermore, experiences of other (health) care professionals in the network and parents (and their children) will be evaluated. Finally, the reach of the new network approach of preventive care for children with overweight and obesity is assessed.

#### Effectiveness study

Effectiveness is measured using three digital questionnaires filled in by parents or caretakers of children with overweight or obesity. The digital questionnaires are sent at baseline (shortly after YHC intake for overweight support), after three months and after one year. Overweight children in four neighbourhoods in ‘s-Hertogenbosch are compared with overweight children in two control municipalities. The focus is on the child’s quality of life, psychosocial problems and parental empowerment. Additionally, differences in outcomes between families with a low and high socio-economic status and between families with/without migration background will be explored.

### Intervention

The theory behind the (new) integrated network approach towards supporting overweight children and their families is based on patient and family empowerment and self-management. To be motivated for a durable behavioural change these families need to increase their autonomy, competences, and connectedness [19]*.*The main intervention within this approach is the new role of the YHC nurse as central care provider. In the four neighbourhoods in ‘s-Hertogenbosch children’s height and weight are measured regularly by professionals who are part of the integrated network approach. The children are measured at YHC contacts but they can also be measured at school or when they visit their general practitioner. In case overweight or obesity is identified at school or by the general practitioner, the child will be referred to a YHC professional. The appointment with the YHC professional will be conducted according to the new guidelines as agreed upon in the network approach of preventive care. YHC nurses received training to fulfil their role as central care provider. The central care provider functions in a network of other professionals who share the vision that overweight is not primarily seen as a consequence of an unhealthy diet and insufficient physical exercise, but rather as a symptom of underlying problems. The central care provider follows six steps. After signalizing overweight (step 1) the central care provider plans a home visit. During this home visit the central care provider explores the underlying causes of overweight by a structured broad interview with parents (step 2). Psycho-education is provided so parents become informed and competent partners. This knowledge increases their autonomy and ability to make choices. In close cooperation with the parents the central care provider makes a plan and the central care provider gets in contact with other involved professionals if needed (step 3 and 4). Collaboration with the social network of the family is encouraged, because it can support the parents and children towards a healthier lifestyle. If needed the central care provider refers the child and/or parents to other professionals or interventions in the medical or social domain (step 5). The central care provider follows the family and coordinates care (step 6).

### Study setting

The integrated network approach of preventive care is implemented in ‘s-Hertogenbosch, a medium-sized city in the Netherlands with 152411 inhabitants [21]. Since 2008, ‘s-Hertogenbosch takes part in a nationally stimulated and coordinated approach called ‘Jongeren Op Gezond Gewicht’ (JOGG, Youth on Healthy Weight). JOGG is an integrated community based approach focussing on locally embedded activities and environmental changes stimulating healthier eating patterns and more physical activity in children [22].

In the effect study children with overweight or obesity in ‘s-Hertogenbosch are compared with overweight or obese children living in one of the control municipalities. These municipalities (nearby ‘s-Hertogenbosch) are Boxtel and Heusden. In these municipalities, the integrated network approach of preventive care for overweight and obese children is not implemented. Overweight and obese children in these municipalities are supposed to receive care as usual according to the guideline for overweight children of the Nederlands Centrum Jeugdgezondheidszorg (NCJ) [20]. We notice that Boxtel and Heusden are smaller than ‘s-Hertogenbosch. No large municipalities without JOGG approach were available in our region to compare with. Characteristics of ‘s-Hertogenbosch, Boxtel and Heusden are described in Table 1.

**Table 1:** Characteristics of inhabitants and children living in intervention and control municipalities [21]

Characteristics	Intervention municipality	Control municipality 1	Control municipality 2
Inhabitants (number)	152411	30655	43516
Children 0–11 years (number)	13023	2644	3734
Dutch background (% children 0–11 years)	78%	85%	88%
Western migration background	8%	8%	7%
Non Western migration background	14%	7%	5%
Education level of the parents			
High education (HBO or university)	53%	36%	35%
Weight status children (1–11 years)			
Overweight	8%	8%	6%
Obese	3%	0%	1%

In ‘s-Hertogenbosch four neighbourhoods participated in the effect and implementation study: Maaspoort, Noord, Zuidoost and West. The characteristics of three of these neighbourhoods were comparable. In Maaspoort inhabitants had a higher mean income, were more often Dutch ethnicity and overweight in children was less common. The implementation of the integrated network approach of preventive care in Maaspoort, Noord and Zuidoost started in 2014 and in West in 2017.

### Study population

#### Implementation study

Youth health care nurses (14), youth health care doctors (8), general practitioners (2), paediatricians (2), dietitian (1), social worker (1), district team worker (1), lifestyle coach (1), project leader (1), manager of the youth health care (1), municipal policy officer (1), program manager of JOGG (1) and parents and overweight children (±10) working or living longer than three months in one of the four intervention areas in ‘s-Hertogenbosch are invited for the interviews. A selection of parents participating in the effect study is also included in the implementation study to share their experiences with the integrated network approach of preventive care. These parents indicated on the informed consent form (filled in for the effect study) that the researchers may contact them for further research. Parents need to have an adequate level of the Dutch language for the interview.

#### Effect study

Children aged 4–12 years old living in one of the four neighbourhoods in ‘s-Hertogenbosch or in one of the control municipalities, are included in the effect study when they are identified as overweight or obese by a YHC professional. Parents should have an adequate level of the Dutch language to complete the questionnaires. Children are excluded from the study when they have severe physical or mental disorders because these could have a large influence on quality of life.

### Study procedure

#### Implementation study

The interviews are conducted by a researcher and a research assistant. The researcher is an YHC doctor, but not in the intervention or control neighbourhoods. The interviews take place at locations chosen by the interviewees. The interviewees sign an informed consent and receive a gift voucher of ten euros for participating.

#### Professionals

The researcher invited eligible professionals in person or via e-mail to participate in the implementation study.

#### Parents and children

The researchers sent an information letter with informed consent to the parents. Several days after sending the information, the researcher contacted the parents to give additional information and answers any questions. If parents agreed on participating in the implementation study, an appointment was made to conduct the interview at a location chosen by the parents.

#### Effect study

Participants are informed about the study by YHC professionals (YHC nurses and doctors) by personal and written information after an appointment in which the child’s weight and health have been discussed. YHC professionals were able to show a short YouTube video about the study to the parents [23]. Thereafter, the YHC professional asked parents whether the researcher may contact them and gave parents an information letter and a form for informed consent. Several days after the appointment, the researcher called the parents to give additional information about the study and to answer any questions. If parents agreed to participate in the study, they were asked to fill in the informed consent and to send it back to the researcher. Finally, a link to the first online questionnaire was sent to the parents by e-mail. Help of a research assistant to fill out the questionnaire was provided if necessary. When parents completed a questionnaire, the child received a small present at the next appointment with the YHC professional. Upon completing all three questionnaires, two free tickets for a swimming pool were sent to the parents and children. If the questionnaire was not filled out in two weeks after sending, a reminder was sent by e-mail. In case the questionnaire was not completed three weeks after sending, the researcher called the parents to ask them to complete the questionnaire. If the parents were unreachable by phone, the request was sent by e-mail.

### Qualitative measurements

#### Interviews with YHC nurses

The individual interviews with the YHC nurses focussed on the extent to which the YHC nurses fulfil their new role as central care providers. In the first part of the interview, open questions were asked regarding the opinion of the YHC nurses about the integrated network approach of preventive care for children with overweight and obesity. Thereafter, the essential tasks of the central care provider were discussed by using a checklist with statements (Table 2). The YHC nurses answered the statements on a 5-point Likert scale, ranging from never (1) to always (5). The total score (range 14–70) quantified/indicated the degree to which the YHC nurses fulfil their tasks as central care provider.

**Table 2:** Essential tasks of the central care provider

Items indicating the extent of implementation of the role of central care provider	
Using the assessment tool	I am using the new assessment tool.
Discussing opportunities	I discuss opportunities that may contribute to a sustainable healthy lifestyle.
Discussing barriers	I discuss barriers that may impede a sustainable healthy lifestyle.
Determining goals in consultation	Together with parents and child, we determine which goals they want to work on.
Connecting to child and parent	I connect as much as possible with the knowledge, possibilities and competences of parents and child.
Involving the social network	I discuss the possibilities of support by the social network of parents and child.
Making a plan of action	Together with parents and child, I make a plan of action.
Dividing tasks	Together with parents and child, I divide tasks.
Coaching the family	I am coaching the families.
Monitoring progress	I monitor the progress of the plan of action and make adjustments when needed.
Informing other professionals	I inform other involved professionals.
Informing the general practitioner	I inform the general practitioner.
Referring parents and children	I refer parents and children to other professionals or interventions if necessary.
Monitoring agreements	I monitor whether agreements are fulfilled.

The second part of the interview focusses on factors which are potentially related to the degree the YHC nurses fulfil their new roles. The questions are based on the measurement instrument for determinants of innovations (MIDI) of Fleuren et al. [24]. This is an instrument to measure the determinants that may affect the implementation of an innovation. The determinants are divided in four categories, namely: characteristics of the user, the innovation, the organisation and the socio-political context. The first three categories of determinants were explored in our study.

#### Interviews with other (health care) professionals

Interviews with other (health care) professionals focussed on their role in the network approach of preventive care for children with overweight and obesity, the (intersectoral) collaboration with other professionals and specific with the central care provider. The interviews started with open questions. The researcher made a drawing of the network partners with whom the professional is in contact. Thereafter, a short questionnaire with eight statements about intersectoral collaboration, adopted from the Quick Scan based on the Development Model for Integrated Care by Minkman (2012), was taken [25]. Besides, some personal information (e.g. age, work experience and perceived workload) was asked. The participant was asked to explain the rates given to the statements.

#### Interviews with parents (and children)

Interviews with parents and children focussed on their experiences in the network approach of preventive care for children with overweight and obesity. Do they appreciate the support from the central care provider and to what extent does the support offered by the network approach really comply with/match their needs?

### Quantitative measurements

#### Questionnaires

##### Demographic characteristics

At baseline, parents were asked about their child’s personal characteristics, such as: name, date of birth, gender, country of birth and living situation. Besides, questions were asked about parents’ country of birth, highest completed education, well-being, perception of their child’s weight status, financial situation, main daily activities and personal goals the family aims to achieve by means of the guidance of the YHC professionals.

##### PedsQL

The Pediatric Quality of Life Inventory (PedsQL) questionnaire was used to measure the quality of life of the child [9, 26–29]. The PedsQL questionnaire consists of 23 items which can be divided in four domains, namely; physical (8 items), emotional (5 items), social (5 items) and school functioning (5 items). Parents answered the questions on 5-point Likert scales ranging from never (0) to always (4). Scores were transformed and the total PedsQL score was calculated by adding up the scores of the four domains. Parent proxy-reported scores for healthy children are on average 83, for overweight children 80 and for obese children 75 [29].

##### IWQOL

The Impact of Weight on Quality of Life (IWQOL) questionnaire was used to measure the quality of life of children with obesity [30]. The IWQOL consists of 27 questions, which are divided in four categories, namely: physical comfort (6 items), body esteem (9 items), social life (6 items) and family relations (6 items). Parents answered the questions on 5-point Likert-scales ranging from always (1) to never (5).

##### SDQ

The child’s psychosocial problems and skills were measured with the Dutch version of the Strengths and Difficulties Questionnaire (SDQ) [31–33]. The SDQ consists of 25 questions, which can be divided in five categories, namely: emotional problems (5 items), conduct problems (5 items), hyperactivity (5 items), peer relationship problems (5 items) and prosocial behaviour (5 items). Questions were answered on a 3-point Likert scale ranging from not true (0) to absolutely true (3). The total SDQ score was calculated by adding up the scores of the first four categories (excluding the score of prosocial behaviour). Cut-off points for the parent-proxy reported SDQ scores are age-dependent. The cut-off points are 15, 14 and 12 for children aged respectively 4–7, 8–11 and 12–14 years old. Scores above the cut-off points indicate potential psychosocial problems [34].

##### EMPO

The empowerment questionnaire (EMPO) for parents was used to measure the parental empowerment in raising children [35]. The questionnaire consists of 12 items, which can be divided in three categories, namely: intrapersonal (4 items), interactional (5 items) and behavioural (3 items). Questions were answered on a 5-point Likert scale ranging from strongly disagree (1) to strongly agree (5). The total EMPO score is the average of the scores on the 12 items. An EMPO score of higher than 3 indicates that parents have sufficient empowerment to raise children [35].

#### Self-efficacy parents

The self-efficacy of parents regarding nutrition and physical activity was measured using 12 statements. These statements were based on the Parental Self-Efficacy for Promoting Healthy Physical Activity and Dietary Behaviours in Children Scale (PSEPAD) [36]. The statements were answered on a scale from 1 (strongly disagree) to 10 (strongly agree).

#### Physical activity and screen time

The physical activity of the child was measured with 12 questions about physical activity and screen time. These questions were derived from questionnaires used by regional public health services in the Netherlands to monitor the health of children. Parents were asked how many days in the week the child was physically active (4 items) and how many days the child spent time behind a screen (2 items). Thereafter, parents were asked to indicate how much time (on such a day) the child spent on the different activities (6 items).

#### Dietary behaviours

The dietary behaviours of the child were measured with 8 questions. These questions comprise how many days a week the child consumes breakfast, fruits, vegetables, warm meals, sweet drinks, snacks and glasses of water. The questions were based on the Health monitor questionnaire of the regional public health services.

#### Biomedical parameters

YHC professionals measured the child’s weight and height during regular check-ups when children are five and nine years old. If the YHC professional identified overweight or obesity, extra appointments were made to monitor the weight of the child. In addition, in the pilot neighbourhoods in ‘s-Hertogenbosch, children were measured annually at several schools where the population of overweight children is high. These measurements were entered in the electronic child file, which automatically calculated the BMI of the child. Overweight and obesity were defined using international age- and gender specific BMI cut-off points for overweight and obesity [20]. The researcher subtracted the BMI of the participating children from their files, at the available time moments, preferably around the time the questionnaires are filled in.

### Analysis

Interviews were analysed with the Atlas.ti (version 7 and 8) software program for qualitative data analysis. All interviews were audio-recorded and transcribed verbatim. Interviews were coded thematically and openly. Several interview transcriptions were coded by two coders independently. Discrepancies between two coders were discussed. Quantitative data were analysed using SPSS Statistics for Windows, Version 21.0 (IBM Corp. Armonk, NY). The characteristics of children and parents in ‘s-Hertogenbosch were compared with the characteristics of children and parents in the control municipalities (means with standard deviation or numbers with percentage). Differences in characteristics and baseline scores on outcome variables (PedsQL, IWQOL, SDQ, EMPO, physical activity questionnaire and BMI) were tested with t-tests for continuous data and Chi-squared tests for categorical data. Multivariate regression models were used to analyse the effect of the intervention on developments in outcome over time (3 months and after one year). We used logistic regression to analyse whether clinical improvement in the primary outcome (+ 4.5 points total PedsQL score) was associated with intervention or control ‘treatment’. In the multivariate models, we also explored potential (confounding or modifying) effects of socioeconomic status (SES: level of parental education and/or income difficulties) or migration background. *P*-values of *p* ≤ 0.05 were considered significant.

### Sample size calculations

A power analysis was performed to calculate the minimum sample size of the effect study to detect a significant effect on children’s quality of life using the PedsQL total score. Previous research conducted in the Netherlands indicated that a change of 4.5 points in the total PedsQL score was clinically relevant [28]. For a difference of 4.5 points in quality of life, with 80% power, 5% significance, a standard deviation of 10 points and a sampling ratio of two, a sample size of 118 children in the intervention group and 59 children in the control group is required.

In the four intervention neighbourhoods in ‘s-Hertogenbosch, more than 700 children between 4 and 12 years old are overweight or obese. A participation rate of 17% is required in order to include 120 children in the study. In the control municipalities, there are fewer children with overweight and obesity. Approximately 450 overweight and obese children are living in these municipalities. If 13% is willing to participate, 60 children can be included in the study. Although inclusion of this population will be challenging, the numbers seem feasible.

## Discussion

The aim of this study is to evaluate the implementation and effectiveness of the integrated network approach of preventive care for children with overweight and obesity. YHC nurses fulfil the role of central care provider in the multidisciplinary network approach. The YHC nurses see children at a regular base and are the linking pins in the social and medical domain. It is expected that with the new integrated network approach more children and parents will be reached more timely and followed up more actively. Moreover, by matching the care with the needs of parents and children and through the optimal use of strong local networks, we expect to achieve durable effects on quality of life for overweight and obese children.

An earlier study in Amsterdam showed that the general practitioners found that they were not able to fulfil the role of central care provider and the professionals opted that YHC nurses were more suited for the role of central care providers [17]. Another small study in Amsterdam showed that YHC nurses seem to be able to fulfil this role. Although developing new competences is important for the YHC nurses, such as involving other professionals in the network [37]. This study in ‘s-Hertogenbosch will show on a larger scale if and how YHC nurses can fulfil the role as central care provider and which aspects could be further improved. We also study if the new approach is already effective on the quality of life of overweight children. With the results of this study, we can optimize the support for overweight children and their parents. The first results are expected to be available in 2019.

### Strengths and limitations

The major strength of this study is the use of both quantitative and qualitative data (triangulation), providing detailed information on both the implementation process and the effectiveness of the integrated network approach of preventive care, which will increase the internal validity of the study.

#### Implementation study

The main strength of the implementation study is that the interview questions for YHC nurses are based on the MIDI framework, a theoretical framework which has been used to concretize the implementation of several healthcare interventions (38). Second, the interviews are held by the researcher who is also an YHC doctor. The researcher is aware of developments in the field of the youth health care and is therefore able to ask more in-depth questions about specific situations. Besides the researcher knew most of the YHC professionals. Therefore, YHC professionals may feel more comfortable to answer the questions truthfully. However, despite the explanation about the research position of the YHC doctor, this could have influenced the responses of the interviewees. Despite stressing that the results of the interview are treated confidentially, some social desirable answers cannot be excluded.

#### Effect study

The main strength of the effect study is that overweight children under support of a central care provider are followed for a year, enabling us to study the medium-term effects of the new integrated network approach of preventive care. Second, validated questionnaires are used to obtain information about the child’s quality of life, psychosocial problems and parental empowerment. These validated questionnaires are filled in by parents or caretakers of the children. Although a child-self report questionnaire of the PedsQL is available for children aged eight years or older, we chose to only include the parent proxy-report of the quality of life of the child, because the group of children aged eight years or older is too small. Besides, the correlation between parents’ proxy reports of their children’s quality of life and children’s self-reported quality of life are fair [27].

Third, the use of a control group increases the internal validity of the study. Though there are some limitations regarding the control group. Due to practical concerns the participants are not randomly assigned to either the intervention- or control group. Besides, since childhood overweight and obesity are high priority for most municipalities, local initiatives to reduce childhood overweight and obesity have been implemented in the control municipalities of Heusden and Boxtel as well. In Heusden an intensive combined lifestyle intervention is started, where children are guided and followed for two years. In Boxtel a community based approach (KANS) is launched to reduce childhood overweight. KANS is a collaboration between schools, lifestyle coaches, family coaches, psychologists, physiotherapists, dieticians, the public health service and the municipality. In addition, at some schools in Boxtel children are measured and weighed annually by a community sports coach. Due to these local initiatives, no ‘clean’ control group is available in the real life setting. Due to this limitation, the differences between the intervention- and control group are expected to be smaller. Nationally, eight municipalities developed and introduced together a model for the integrated network approach of preventive care in 2018 [38]. In the next coming years an integrated network approach of preventive care for overweight children will be implemented in more Dutch municipalities.

## Data Availability

The questionnaires and interview structures are available in Dutch from the corresponding author upon reasonable request. The datasets generated and analysed during the current study are not publicly available but are available from the corresponding author on reasonable request.

## Abbreviations

BMI: Body Mass Index
CCP: Central Care Provider
EMPO: Empowerment Questionnaire
IWQOL: Impact of Weight on Quality of Life
JOGG: Jongeren Op Gezond Gewicht (Youth on healthy weight)
METC: Medical Ethics Committee
MIDI: Measurement instrument for determinants of innovations
NCJ: Nederlands Centrum Jeugdgezondheidszorg
PedsQL: Pediatric Quality of Life Inventory
PSEPAD: Physical Activity and Dietary Behaviours in Children Scale
SDQ: Strengths and Difficulties Questionnaire
SES: Socioeconomic Status
YHC: Youth Health Care
